# The influence of non-stationarity of spike signals on decoding performance in intracortical brain-computer interface: a simulation study

**DOI:** 10.3389/fncom.2023.1135783

**Published:** 2023-05-12

**Authors:** Zijun Wan, Tengjun Liu, Xingchen Ran, Pengfu Liu, Weidong Chen, Shaomin Zhang

**Affiliations:** ^1^Key Laboratory of Biomedical Engineering of Education Ministry, Zhejiang Provincial Key Laboratory of Cardio-Cerebral Vascular Detection Technology and Medicinal Effectiveness Appraisal, Department of Biomedical Engineering, School of Biomedical Engineering and Instrument Science, Zhejiang University, Hangzhou, China; ^2^Qiushi Academy for Advanced Studies, Zhejiang University, Hangzhou, China

**Keywords:** brain-computer interfaces, simulation, chronic intracortical recording, recurrent neural network, training scheme

## Abstract

**Introduction:**

Intracortical Brain-Computer Interfaces (iBCI) establish a new pathway to restore motor functions in individuals with paralysis by interfacing directly with the brain to translate movement intention into action. However, the development of iBCI applications is hindered by the non-stationarity of neural signals induced by the recording degradation and neuronal property variance. Many iBCI decoders were developed to overcome this non-stationarity, but its effect on decoding performance remains largely unknown, posing a critical challenge for the practical application of iBCI.

**Methods:**

To improve our understanding on the effect of non-stationarity, we conducted a 2D-cursor simulation study to examine the influence of various types of non-stationarities. Concentrating on spike signal changes in chronic intracortical recording, we used the following three metrics to simulate the non-stationarity: mean firing rate (MFR), number of isolated units (NIU), and neural preferred directions (PDs). MFR and NIU were decreased to simulate the recording degradation while PDs were changed to simulate the neuronal property variance. Performance evaluation based on simulation data was then conducted on three decoders and two different training schemes. Optimal Linear Estimation (OLE), Kalman Filter (KF), and Recurrent Neural Network (RNN) were implemented as decoders and trained using static and retrained schemes.

**Results:**

In our evaluation, RNN decoder and retrained scheme showed consistent better performance under small recording degradation. However, the serious signal degradation would cause significant performance to drop eventually. On the other hand, RNN performs significantly better than the other two decoders in decoding simulated non-stationary spike signals, and the retrained scheme maintains the decoders’ high performance when changes are limited to PDs.

**Discussion:**

Our simulation work demonstrates the effects of neural signal non-stationarity on decoding performance and serves as a reference for selecting decoders and training schemes in chronic iBCI. Our result suggests that comparing to KF and OLE, RNN has better or equivalent performance using both training schemes. Performance of decoders under static scheme is influenced by recording degradation and neuronal property variation while decoders under retrained scheme are only influenced by the former one.

## Introduction

Intracortical Brain-Computer Interfaces (iBCI) have the potential to restore motor function in paralyzed patients (Hochberg et al., 2012; Aflalo et al., 2015; Salahuddin and Gao, 2021; Willett et al., 2021). Recent iBCI studies were able to achieve fluent control of cursors and prostheses using subjects’ neural signals, such as spikes or local field potentials (LFPs) (Wodlinger et al., 2015; Pandarinath et al., 2017). However, despite the remarkable achievements of iBCI studies, many more challenges remain (Gilja et al., 2011; Rapeaux and Constandinou, 2021). One of such challenge is the non-stationarity of neural signals in chronic intracortical recording (Carmena et al., 2005; Wang et al., 2014; Downey et al., 2018), which involves many complicated factors and is thus difficult to evaluate. A typical cause of non-stationarity is the glial scarring induced by immune rejection (Salahuddin and Gao, 2021). Glial cells could wrap the tips of intracortical microelectrode due to foreign body reaction during recordings, leading to a decrease in the peak amplitude of spike signals (Polikov et al., 2005; Rapeaux and Constandinou, 2021). Likewise, the neuronal firing patterns of subjects could adapt to fit the patterns of decoders, leading to intrinsic changes in neurons (Jarosiewicz et al., 2008; Ganguly and Carmena, 2009; Orsborn et al., 2014; Takmakov et al., 2015). These degradations and neuronal variances will increase the non-stationarity of neural signals.

Previous studies mainly used mean firing rate (MFR) and number of isolated neurons (NIU) to describe recording degradation while neural preferred direction (PDs) was used to describe neural signal variance (Simeral et al., 2011; Ahmadi et al., 2021). Previous chronic iBCI studies have reported a decrease in MFR (Wang et al., 2014; Ahmadi et al., 2021) and NIU as recording time increases (Rousche and Normann, 1998; Sharma et al., 2015; Ahmadi et al., 2021), as well as time-varying changes in neural PDs (Ganguly and Carmena, 2009; Fraser and Schwartz, 2012; Wang et al., 2014). Overall, these above results suggest that the non-stationarity of neural signal has a great influence on iBCI performance and presents a great challenge to its practical application. To overcome this challenge, lots of decoders and various training schemes have been applied to achieve better performance (Jarosiewicz et al., 2013; Ahmadi et al., 2021), but it is still unclear how a single type of non-stationarity could affect the performance of decoders.

Generally, motor iBCI decoders establish the statistical model mapping neural signals to their corresponding kinematic signals (Velliste et al., 2008; Gilja et al., 2012; Hochberg et al., 2012; Li et al., 2012; Flint et al., 2013; Inoue et al., 2018; Willett et al., 2021). Researchers have implemented several high-performance decoders. The following three widely used decoders are chosen for this study to evaluate the effects of non-stationarity variations on decoding performance: (1) Optimal Linear Estimation (OLE) (Chase et al., 2009), which is based on the linear model optimized by the ordinary least square estimator. It gives predictions directly using linear mapping functions from neural signal to kinematic signal; (2) Kalman filter (KF) (Gilja et al., 2012; Hochberg et al., 2012; Pandarinath et al., 2017), which works by a two-phase process. It produces estimates of the current kinematic signals in the prediction phase. It updates the predictions using a weight average between the kinematic signals in the prediction phase and the kinematic signal estimated with the observed neural signals; and (3) Recurrent Neural Network (RNN) (Sussillo et al., 2012; Zaremba et al., 2014), which is based on a non-linear model that predicts kinematic signals using the sequential information of input neural signals. Due to its unique structure, RNN was reported to have better performance than KF and OLE in some sequential decoding tasks (Sussillo et al., 2012).

Apart from their implementation, training schemes can also influence the performance of decoders. Static scheme and retrained scheme were both investigated in this study. In the static scheme, decoders are trained with data in the first session and the parameters were kept fixed in the rest sessions (Jarosiewicz et al., 2013; Ahmadi et al., 2021). Decoders under static scheme are easy and convenient to deploy but are less resistant to non-stationarity in neural signals. In the retrained scheme, decoders are retrained with data in each session and then tested with data in the same session. Thus, they often have increased performance comparing to decoders under static scheme (Jarosiewicz et al., 2013; Milekovic et al., 2018). It will be practically advantageous if we know approximately how much the variations of each type of non-stationarity influences the decoding performance with different training schemes, because we could choose suitable decoders and training schemes to maintain robust performance in chronic iBCI experiments based on the findings.

Since recording degradation and neuronal variance usually change simultaneously in real-world iBCI experiments, it is difficult to investigate the effects of each type of non-stationarity specifically using recording data. Therefore, neural simulation was used to synthesize spike data so that different types of non-stationarity can be introduced separately (Kim et al., 2018). The simulation model is not only used in offline spike generation (Chase et al., 2009; Inoue et al., 2018; Wan et al., 2019) but also adopted to build online spike signal simulators (Awasthi et al., 2022; Ferrea et al., 2022). It reduced experimental expenses in close-loop experiments and utilized the full advantage of previous data. One of the most commonly used spike simulation methods is the one based on the Population Vector (PV) model (Georgopoulos et al., 1986). The PV model comes from the observation of physiological 2D center-out experiments and is explainable from the neuroscience perspective, which is suitable for the implementation of non-stationarity variations. In this study, we generated the spike data with the PV model using 2D kinematic data recorded from a real iBCI center-out experiment.

Our results demonstrate that: (1) RNN performs better or equivalent in comparison with that of KF and OLE when using either training scheme. (2) Performance of decoders trained with both schemes are influenced by recording degradation and decoders under static scheme are also influenced by neural variation.

## Materials and methods

**FIGURE 1:**
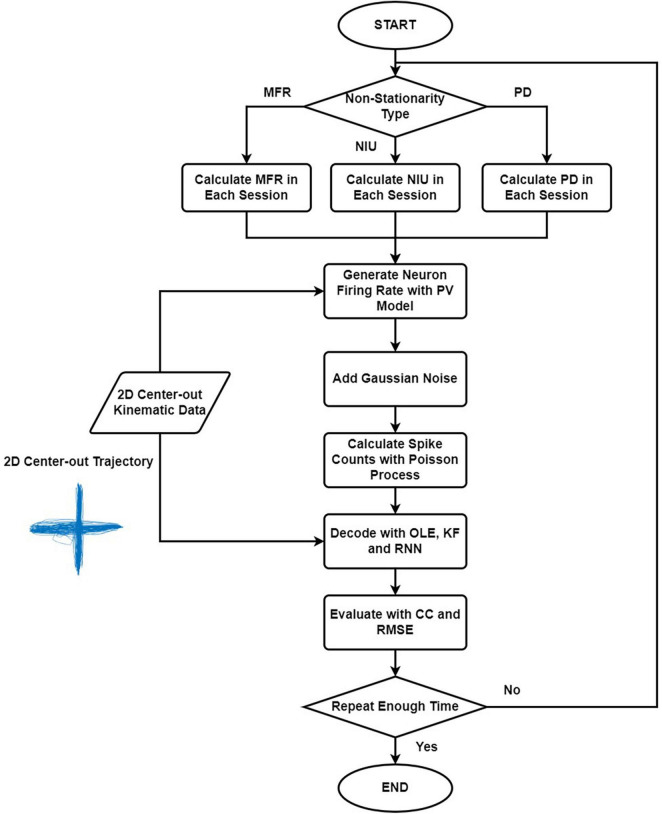
The flow chart explaining the simulation and decoding procedure. Firstly, we selected the non-stationarity metric type to simulate. Then we generated the corresponding parameters, and the spike signals were simulated with the PV model using the 2D kinematic data. Next, we estimated the spike counts using Poisson process. Lastly, we trained and tested the decoders with simulated spike data and evaluated the results. The procedure was repeated 20 times.

Figure 1 shows the simulation and decoding procedure. For simulation part, we selected the non-stationarity metric type and generated the corresponding parameters. Then, we simulated the spike signal with the PV model using the 2D kinematic data, and we estimated the spike counts with Poisson process. For decoding part, we trained and tested the decoders with simulated spike data and evaluated the results. The procedure was repeated 20 times. The decoding results of the simulated spikes were used to evaluate the influences of different types of non-stationarity.

### Parameters setting for simulating the non-stationarity of neural signals in chronic recordings

As reported by several studies, recording degradation and neuronal variance are the main source of neural signal non-stationarity in chronic intracortical recordings (Chestek et al., 2011; Wang et al., 2014). Here, we used three metrics (MFR, NIU, and PDs) to imitate the non-stationarities of neural signals in chronic recordings. MFR decrease and NIU loss were used to simulate the recording degradation and PD change to simulate the neuronal variance. The parameters of MFR, NIU, and PDs were changed in each session of all simulations.

In each type of non-stationarity experiment, we simulated 11 5-min sessions of spike data with different non-stationarity variances. A total of 3,000 sample points were provided with a sample rate of 10 Hz in each session.

#### MFR decrease

**FIGURE 2:**
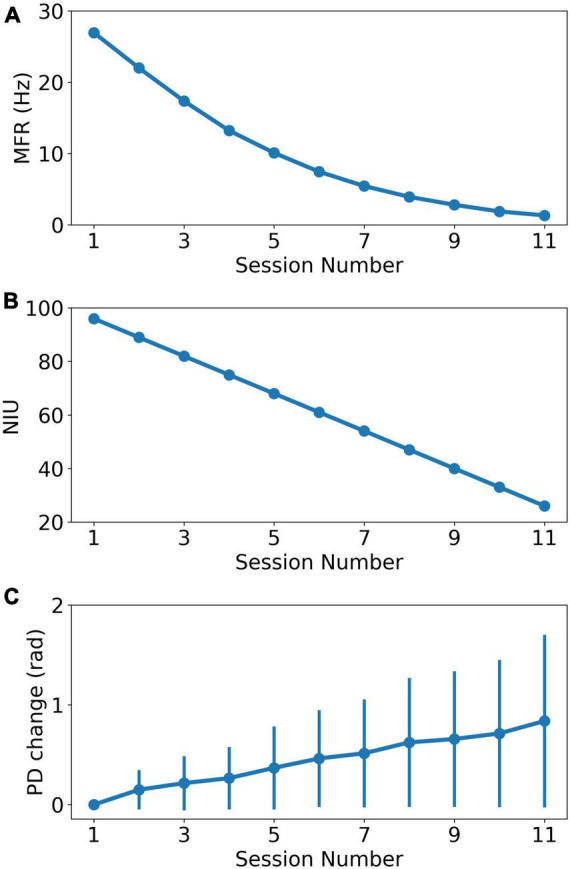
The change of recording properties as the recording session index increases. **(A)** The MFR decreasing curve as the recording session increases. **(B)** The NIU dropping curve as the recording session increases. **(C)** The average PD change curve based on curve fitting model. The error bar represents the standard error of PDs change relative to PDs in the first session.

Previous studies have reported an approximately negatively linear relationship between neurons’ MFR and recording time (Downey et al., 2018; Ahmadi et al., 2021), which aligns with the chronic intracortical recording data collected in our previous work (Wang et al., 2014). Therefore, MFR was assumed to decrease linearly in our simulation. Specifically, two steps were involved in spike data generation. We first generated spike data for all sessions. Some spike counts were then dropped to mimic the aforementioned degradation, shown in Eq (1).


(1) $$\begin{array}{c} f_{i}\,\left(n\right)=f_{i}\,\left(1\right)-d_{i},d_{i}∼N\,\left(n*r,\sigma \right), \end{array}$$

where *i*,*n*,*r* are the neuron index, session index and scale ratio, respectively. *f*_*i*_(*n*) is the MFR of neuron *i* in session *n*. *d_i_* is the number of dropped spikes sampled from a Gaussian distribution with mean equal to n*r. The minimum firing rate was set to 0 for each neuron. We decreased the MFR from 28.0 Hz to about 1.0 Hz in the 11 sessions. We calculated the firing rate of real recorded neural data and it shows that firing rates of more than 90% neurons are lower than 28 Hz, shown in Supplementary Figure 1. Based on previous reports indicating that spike signals with extremely lower firing rates could not carry enough effective information (Wang et al., 2014), the minimum firing rate of the last session was set to 1 Hz instead of 0. Figure 2A shows the MFR decreased tendency as the recording session index increases.

#### NIU loss

Similar to MFR, NIU loss is also approximately negatively linear to recording time (Ahmadi et al., 2021). In addition, our experiment data showed a trend of linearly decrease in the number of detective units in chronic intracortical recording (Wang et al., 2014). In this study, a linear model was used to simulate NIU loss to simplify the research conditions. The simulation of NIU loss can be divided into two steps: Firstly, we calculated the NIU in each session based on the linear model, which ranged from 96 in the first session to 26 in the last session (Wang et al., 2014). Secondly, we generated the spike counts of remaining neurons in each session. In each session, the firing rate was set to 0 for the neurons that were dropped, which means no spike firing was collected from the dropped neurons (Degenhart et al., 2020). Figure 2B shows the NIU in each session.

#### PD changes

In chronic recording, it is reported that the neuron PD varied in different sessions (Dickey et al., 2009; Fraser and Schwartz, 2012), which is also confirmed by our own data (Wang et al., 2014). The PD variations are affected by a wide variety of factors, but can be fit approximately using the following curve function (Fraser and Schwartz, 2012):


(2) $$\begin{array}{c} y\,\left(n\right)=b_{1}-b_{2}\,e\,x\,p\,\left(b_{3}*n\right)+\varepsilon \,\left(n\right), \end{array}$$

where *n*,*b* are session index and model parameters, respectively. y(n) is the predicted change of PD in session n. ε(*n*) is the noise in session *n*. The change in PD is defined as the difference between PD in the first session and PD in current session. This model will be henceforth referred to as the curve fitting model. The simulation contains two steps. Individual PD changes were first calculated for each neuron in each session, with changes ranging from 0 to 0.8 rad. The individual changes were then added to the initial PDs as the sessions continue, forming the PDs change in each session. Then, spike counts were generated with corresponding parameters in each session. Figure 2C shows the PDs change in each session.

### Spike activity simulation

A simulation method based on the PV model was applied (Georgopoulos et al., 1986; Inoue et al., 2018). In this model, the spike firing rates are synthesized with speed signal, shown in Eq (3).


(3) $$\begin{array}{c} f_{i}\,\left(t\right)=b_{i,0}+b_{i,1}\,{||v\,\left(t\right)||}_{2}\,c\,o\,s\,\left(\theta \,\left(t\right)-\theta_{P\,D}\right)+b_{i,s}\,{||v\,\left(t\right)||}_{2}+\varepsilon \,\left(t\right), \end{array}$$

where *b*_*i,0*_ is the baseline firing rate of neuron *i*. *b*_*i,1*_ and θ_*PD*_ are the modulation depth and preferred direction of neuron *i*. *b*_*i,s*_ describes the speed modulation of neuron *i*. *t* is the sample time point. The sample rate of firing rate is the same as that of kinematic signal, which is 10 Hz. ||*v*(*t*)||_2_ is the second-order norm of speed at time *t*. ε(*t*) is the noise representing the deviation from the model. *f*_*i*_(*t*) is the firing rate of neuron *i* at time *t*.

The baseline firing rate *b_0_* of all neurons were sampled from a normal distribution with a mean of 20 Hz and a variance of 6 based on the recorded spike data in our previous study (Wang et al., 2014). The modulation depth *b*_*i, 1*_ and speed factor *b*_*i, s*_ of all neurons obeyed the normal distribution with a mean of 5 and a variance of 2. θ_*PD*_ obeyed a uniform distribution on [0, 2*π]. After generating the neural firing rate with Eq (3), we estimated the spike counts in each time bin. We assumed every detection of spike signal as a Bernoulli trial, so the binned spike counts obey the Poisson distribution. Therefore, the Poisson Process shown in Eq (4) was applied to probabilistically generate the binned spike counts (Inoue et al., 2018; Kim et al., 2018).


(4) $$\begin{array}{c} \begin{array}{c} P\left(N\left(t+\tau \right)-N\left(t\right)=k\right)=\frac{{\left(\tau \,\lambda \right)}^{k}\,e^{-\tau \,\lambda }}{k!} \end{array} \end{array}$$

where τ=0.1 s is the time interval and λ is the expected value of the Poisson distribution *N*. The left side of Eq (4) is the probability of a neuron firing *k* times between time *t* and *t* + τ. According to Eq (4), the binned spike counts can be estimated by using λ=*f*_*i*_(*t*), where *f*_*i*_(*t*) is derived from Eq (3).

According to Eq (3), spike simulation needs speed signals as input. The speed signals in our study were collected from a kinematic dataset from one of our previous monkey studies (Li et al., 2014; Wang et al., 2014), in which, a monkey was trained to control a cursor on screen using a joystick in a center-out task. At the start of each trial, a cursor was shown at the center of the screen, as a target appeared randomly in one of the four positions around the screen (top, bottom, left, right). The monkey was trained to move the cursor to reach the target and held it steadily within a limited time. Then, the monkey was required to move the cursor back to center position. Upon a successful attempt, the monkey would receive water as reward. No reward was given otherwise. In this study, the speed signals were calculated from the cursor trajectory and normalized with a z-score before simulation. The example cursor trajectory is shown in Figure 1. We used two sets of cursor trajectories from the 2D center-out task conducted by a monkey. The first set of trajectory data was used to simulate spikes in the first session. For comparison, we used the second trajectory set repetitively to simulate spikes in the latter 10 sessions.

### Decoders

Optimal Linear Estimation, KF, and RNN were used as decoders to investigate the effect of each type of non-stationarity on decoding performance.

#### Optimal linear estimation

Optimal linear estimation is an improvement to the Population Vector algorithm (PVA). In OLE, the encoding equation can be written as:


(5) $$\begin{array}{c} r_{t}=B\,v_{t}+\varepsilon_{t}, \end{array}$$

where *r_t_* is an n*1 vector consisting of the firing rates of sampled neurons. n is the number of neurons. *B* is a n*3 matrix consisting of the preferred directions of sampled neurons and baseline firing rates. *v_t_* is the velocity vector, and ε_*t*_ is the measurement error. In OLE, the matrix *B* is used as the explanatory variable in the multiple regression model, the predicted velocity vector ${\tilde{v}}_{t}$ can thus be calculated as:


(6) $$\begin{array}{c} {\tilde{v}}_{t}={\left(B^{{}^{\prime }}\,B\right)}^{-1}\,B^{{}^{\prime }}\,r_{t}, \end{array}$$

In OLE, the multiple regression model is used to reduce the decoding error caused by the non-uniformity of PD of neurons. The speed gain in each movement direction obeys uniform distribution. If *B*′*B* = *I*, where *I* is the identity matrix, then the preferred directions of sampled neurons are uniformly distributed. OLE and PVA have the same performance under this condition. There are 288 parameters in our OLE model.

#### Kalman filter

As one of the commonly used decoders in motor neural signal decoding, KF (Welch and Bishop, 1995) illustrates the observed spike signals changing over time with a first-order Markov model defined in Eq (7a). Where *v_t_* is the kinematic signals at time t. A is the state transform matrix. δ_*t*_ is the Gaussian noise with zero mean and variance *Q*. KF also assumes a linear relationship between the spike signals and the kinematic signals, as defined in Eq (7b), where *r_t_* is the spike signals. *H* is the transform matrix. ε_*t*_is the Gaussian noise with zero mean and variance *R*. *H* and *A* are estimated by the least square method in model calibration. In each iteration, we predicted the priori kinematic data *v_t_* using the first-order Markov model in Eq (7a) and the linear model in Eq (7b). Then, we calculated the optimal Kalman gain and updated the posterior estimation of the kinematic data (Wang et al., 2014). There are 9,420 parameters in this model.


(7a) $$\begin{array}{c} v_{t}=A\,v_{t-1}+\delta_{t},\delta_{t}∼N\,\left(0,Q\right), \end{array}$$

(7b) $$\begin{array}{c} r_{t}=H\,v_{t}+\varepsilon_{t},\varepsilon_{t}∼N\,\left(0,R\right), \end{array}$$

#### Recurrent neural network

Recurrent neural network is widely used for its excellent ability to predict sequential signals (Sussillo et al., 2012, 2016). In this study, a vanilla RNN was used to decode the simulation signals. It took the spike counts of all neurons in a bin time as input, then output the predicted speed at the bin time. Our RNN implementation can be defined as follows:


(8a) $$\begin{array}{c} h_{t}=W_{i\,n}\,r_{t}+b_{i\,n}, \end{array}$$

(8b) $$\begin{array}{c} o_{t}=f\,\left(h_{t}+W_{h}\,h_{t-1}\right), \end{array}$$

(8c) $$\begin{array}{c} {\tilde{v}}_{t}=W_{o}\,o_{t}+b_{o}, \end{array}$$

where *r_t_*, *h_t_*, *o_t_*, ${\tilde{v}}_{t}$ each represents the input neuron signal, the input of hidden layer value, the output of hidden layer and the predicted velocity vector. *W*_*in*_, *W_h_*, and *W_o_* are the weights of the input layer, the recurrent layer, and the output layer. *b*_*in*_ and *b_o_* are the bias in the input layer and output layer. The Hessian-Free (HF) optimization was used to optimize the network parameters (Martens, 2010). HF is a second-order algorithm for calculating the gradient descent in back-propagation. To avoid the intensive calculation required in solving the Hessian matrix, HF uses the conjugate gradient decrease method to estimate the local minimum point with iterations. Compared to the traditional backpropagation through time (BPTT), HF is faster, more accurate and easier to use for finding the optimal solution. HF has four primary parameters: the maximum HF iteration times, the initial damping parameter λ, the minimum and the maximum conjugate gradient iteration times. We also used L2 regularization to reduce overfitting. The mean squared error (MSE) was used as the loss function for this study. A more detailed description of network parameters is shown in Table 1.

**TABLE 1:** The detailed hyperparameters of RNN.

Name	Value	Name	Value
Hidden layer	1	Max HF iteration	1,000
Hidden units	48	Min CG iteration	10
Activation function	tanh	Max CG iteration	100
Drop out	0.5	λ	0.004
L2 weight	0.01	Total parameters	7,106

Hessian-Free (HF) refers to hessian free optimization method. CG is the conjugate gradient, which is used to estimate the optimal gradient descent direction. λ is the damping parameter in HF optimization, and L2 refers to L2 regularization.

### Training schemes

**FIGURE 3:**
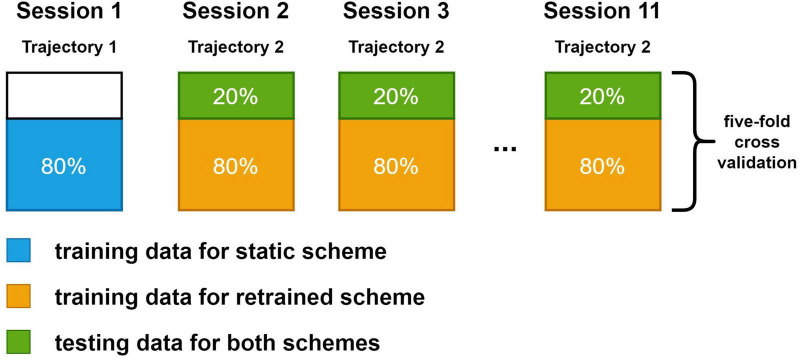
The dataset split method for static and retrained decoding. Each large box represents the data in a session. The blue box and orange box are the training data for static scheme and retraining scheme. Green box is the testing data, which is the same for both schemes. Spike data in the first session were generated with trajectory data in the first set while spike data in the rest sessions were generated with the same trajectory data in the second set.

Two training schemes were investigated in this study. In static scheme, 80% of the data (2,400 sample points) in the first session were used for training. The decoders were trained once and their parameters were fixed in the following sessions. In retrained scheme, decoders were trained in every session. A total of 80% data in the session was used for training. In both schemes, the rest 20% data were used for testing. The test data for both schemes were the same in each session to ensure the comparability of results. We compared results from the second session to the last session since the decoders under static scheme and decoders under retrained scheme were the same in the first session. A more detailed view of the dataset arrangement for both schemes is shown in Figure 3. We used a fivefold cross-validation for all sessions.

### Evaluation

In this study, the decoding performance was evaluated with Pearson’s Correlation Coefficient (CC) and Root Mean Square Error (RMSE). CC represents the similarity of shapes in real and decoded speed, while RMSE represents the average error magnitude between real and decoded speed. The definition of CC and RMSE are as follows:


(9) $$\begin{array}{c} C\,C=\frac{C\,o\,v\,\left(X,\hat{X}\right)}{\sqrt{V\,a\,r\,\left(X\right)*V\,a\,r\,\left(\hat{X}\right)}}, \end{array}$$

(10) $$\begin{array}{c} R\,M\,S\,E=\sqrt{\frac{\left(X-\hat{X}\right)*{\left(X-\hat{X}\right)}^{T}}{N}}, \end{array}$$

where $C\,o\,v\,\left(X,\hat{X}\right)$ is the covariance of real data X and decoded data $\hat{X}$. *Var*(*X*) is the variance of *X*. And *N* is the sample point number of *X* and $\hat{X}$.

### Statistical analysis

To reduce the effects of randomness in the simulation process, the whole simulation procedure was repeated 20 times for each type of non-stationarity experiments. The performance of decoders and schemes was assessed by the CCs and RMSEs of predicted velocity and real velocity. Performance was evaluated for each session in each repetitive simulation experiment. We used the two-way ANCOVA to verify the influence of non-stationarity metrics and decoder types on the decoding results. The significance results are shown in Supplementary Table 1. The main effect of the metrics is significant and Mann-Whitney *U*-test with Bonferroni’s correction was used to test the difference between decoder performance. The significance level α was set to 0.01.

Several boxplots were used to demonstrate the decoder performance in the last session, where the neural non-stationarity was the largest. The horizontal line in the box denotes the medium value, and the box represents the interquartile range (between 25th and 75th percentiles). The whiskers extend 1.5 times of the interquartile range. The diamond points are data points out of the whiskers range. Simulation procedure code was written in MATLAB. The program was running on MATLAB R2020a, with an i5-7400 CPU and 16 GB memory.

## Results

### Decoding performance comparison when MFR decreases

**FIGURE 4:**
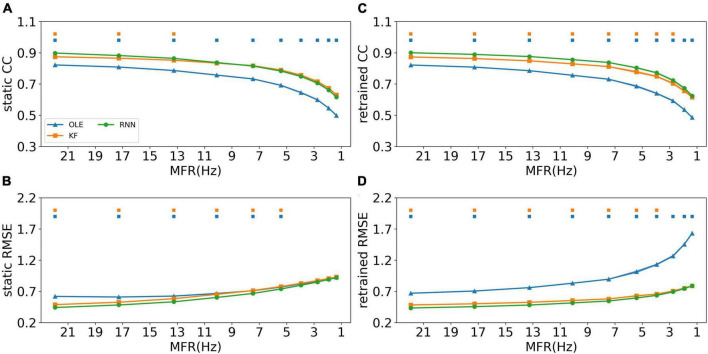
The decoding results of the MFR simulation experiment. **(A)** The CCs of all decoders using static decoding. The *x*-axis is the MFR of all simulated neurons, and the *y*-axis is the value of CC. The blue, orange, and green lines represent OLE, KF, and RNN. The shadows represent the 95% confidence interval of repetitive simulation. A green and/or orange squares is drawn above each session whenever RNN had a significant better performance than OLE, and KF (Mann-Whitney *U*-test with Bonferroni’s correction, *p* < 0.01). **(B)** Similar to **(A)** but for RMSE. **(C,D)** Similar to **(A,B)** but for retraining scheme.

The effects of MFR decline were subsequently examined for all training schemes and decoders. Figure 4 shows the CCs and RMSEs of all decoders using different training schemes. For static decoding (shown in Figures 4A, B), CCs decreased and RMSEs increased as MFR went down, indicating a causal relationship between neuronal MFR decline and performance degradation. RNN outperformed the other two decoders in the first 3 sessions when static scheme was applied (Mann-Whitney *U*-test with Bonferroni’s correction, *p* < 0.01). For decoders under retrained scheme (shown in Figures 4C, D), we can see a performance drop similar to that of the static scheme. As for the decoders, RNN performed similarly to KF when the MFR was quite low, whereas OLE was outperformed by both RNN and KF under all settings.

**FIGURE 5:**
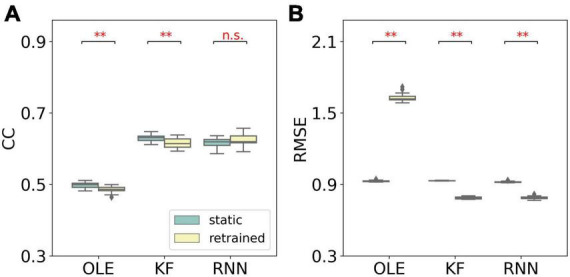
Performance comparison of different training schemes in the last session of the MFR simulation experiment. **(A)** The box plot of CCs of each decoder with static and retrained schemes. Cyan boxes are the CC distributions of decoders under static scheme and yellow boxes are that of decoders under retrained scheme. Mann-Whitney *U*-test with Bonferroni’s correction was used to calculate significance (n.s.: no significance, *p* > 0.05; **p < 0.01). **(B)** Similar to **(A)** but for RMSE.

A performance comparison (shown in Figure 5) was conducted specifically for the last session where MFR was the lowest. Retrained RNN performed better than static RNN in RMSE comparison, and the average CC of retrained RNN is higher than static RNN. Static OLE showed a significantly better performance than retrained OLE (Mann-Whitney *U*-test with Bonferroni’s correction, *p* < 0.01), which might be a result of its linear and straightforward design. These results suggest that retrained RNN has better performance than OLE and KF when the decline of MFR is not severe. Also retrained RNN outperformed static RNN when MFR decreased.

### Decoding performance comparison when NIU decreases

**FIGURE 6:**
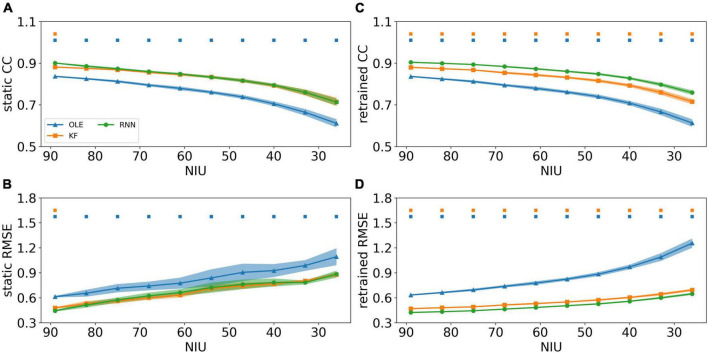
The decoding results of the NIU loss experiment. **(A)** The CCs of all decoders using static decoding. The *x*-axis is the remained NIU, and the *y*-axis is the value of CC. The blue, orange, and green lines represent OLE, KF, and RNN. The shadows represent the 95% confidence interval of repetitive simulation. A green and/or orange squares is drawn above each session whenever RNN had a significant better performance than OLE, and KF (Mann-Whitney *U*-test with Bonferroni’s correction, *p* < 0.01). **(B)** Similar to **(A)** but for RMSE. **(C,D)** Similar to **(A,B)** but for retraining scheme.

To investigate the effects of the NIU loss, spike data were generated and decoded using different decoders and training schemes. Their performance is shown in Figure 6, where Figures 6A, B demonstrates the decoding performance using the static scheme. As shown in the line charts, the CCs decreased while RMSEs increased for all decoders as NIU declined. Using static decoding, RNN consistently outperformed OLE in terms of both CC and RMSE, while offering better capability than KF in the first test session (Mann-Whitney *U*-test with Bonferroni’s correction, *p* < 0.01). However, no significant difference was found between the performance of RNN and KF when the NIU continued to drop. As for retrained decoding shown in Figures 6C, D, the performance of all decoders declined as the NIU decreased, with RNN having the best performance in all sessions (Mann-Whitney *U*-test with Bonferroni’s correction, *p* < 0.01).

**FIGURE 7:**
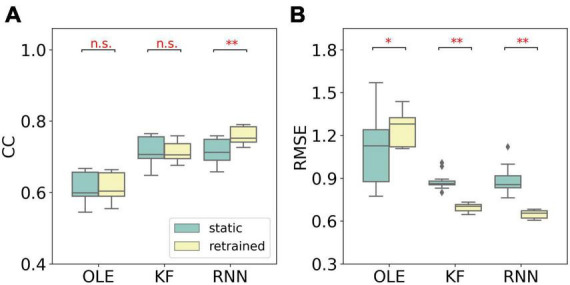
The comparison of decoding performance with different training schemes in the last session in the NIU loss experiment. **(A)** The CC of all decoders with static and retrained schemes. Cyan boxes are the CC distributions of decoders under static scheme and yellow boxes are that of decoders under retrained scheme. Mann-Whitney *U*-test with Bonferroni’s correction was used to calculate the significance (n.s.: no significance, *p* > 0.05; *p < 0.05; ^**^p < 0.01). **(B)** Similar to **(A)** but for RMSE.

Similar to MFR, we compared the performance of different training schemes in the last session, as shown in Figure 7. The retrained RNN and KF outperformed the static version (Mann-Whitney *U*-test with Bonferroni’s correction, *p* < 0.01). For OLE, the static scheme had a lower RMSE than that of the retrained scheme. Our result suggests that retrained RNN has the best performance when non-stationarity is limited to NIU decrease.

### Decoding performance comparison when the PDs change

**FIGURE 8:**
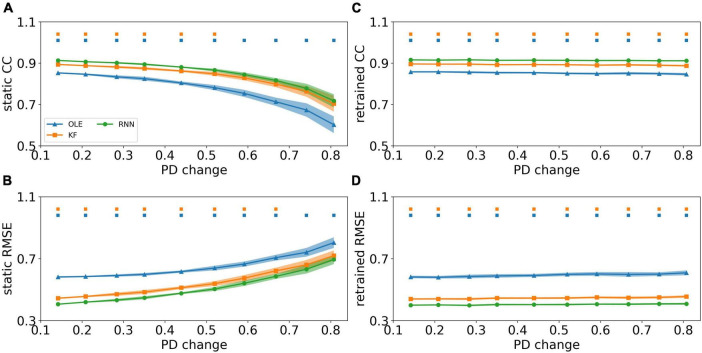
The decoding results of PDs change experiment. **(A)** The CCs of all decoders using static decoding scheme. The *x*-axis is the change of PD, and the *y*-axis is the value of CC. The blue, orange, and green lines represent OLE, KF, and RNN. The shadows represent the 95% confidence interval of repetitive simulation. A green and/or orange squares is drawn above each session whenever RNN had a significant better performance than OLE, and KF (Mann-Whitney *U*-test with Bonferroni’s correction, *p* < 0.01). **(B)** Similar to **(A)** but for RMSE. **(C,D)** Similar to **(A,B)** but for retrained decoding.

We investigated the effect of PDs change based on the curve-fitting model. Figures 8A, B showed a performance drop in all decoders as the change of PDs got larger. RNN outperformed the other decoders in test sessions with a small PDs change in the early sessions (Mann-Whitney *U*-test with Bonferroni’s correction, *p* < 0.01) and performed similarly to KF in the latter sessions (Mann-Whitney *U*-test with Bonferroni’s correction, *p* > 0.01). Figures 8C, D showed the CCs and RMSEs of decoders with retrained scheme, where the performance of all decoders remained at a relatively stable level regardless of changes in PDs. RNN outperformed the other two decoders in all test sessions (Mann-Whitney *U*-test with Bonferroni’s correction, *p* < 0.01), while KF maintained as a close second (Mann-Whitney *U*-test with Bonferroni’s correction, *p* < 0.01).

**FIGURE 9:**
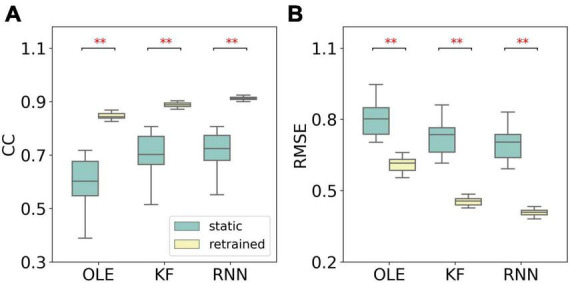
The comparison of decoding performance with different decoding training schemes in the last session in the PDs experiment. **(A)** The CCs of all decoders with static and retrained schemes. Cyan boxes are the CC distributions of decoders under static scheme and yellow boxes are that of decoders under retrained scheme. Mann-Whitney *U*-test with Bonferroni’s correction is used to calculate the significance (n.s.: no significance, *p* > 0.05; ^**^p < 0.01). **(B)** Similar to **(A)** but for RMSE.

Figure 9 compares the performance of different training schemes in the last session. Noticeably, all decoders under static scheme performed significantly worse than decoders under retrained scheme (Mann-Whitney *U*-test with Bonferroni’s correction, *p* < 0.01), suggesting trivial influence of PDs variances on the performance of retrained decoding.

## Discussion

In this study, we investigated how different types of non-stationarity influence the decoding performance of different decoders and training schemes. The effects of three types of non-stationarity, either caused by recording degradation (MFR decline, NIU loss) or neuronal property variance (PDs variation), were evaluated using three decoders and two training schemes. Although conducted under hypothetical settings, our simulation provides a new aspect for the examination of signal degradation. Both linear and non-linear decoders, as well as different training schemes were compared in this study. Our result suggests that neural signal degradation leads to a performance decrease in all three decoders (OLE, KF, and RNN) and both training schemes. Overall, decoders under retrained scheme kept stable performance regardless of variance increased. RNN also demonstrated better or equivalent performance consistently when compared with KF and OLE.

### The influence of degradation and neuronal property variance on chronic decoding

It has been reported that the variation of neuronal properties has effects on decoding performance (Kim et al., 2018). Here we further investigated the influence of spike signal non-stationarity by considering both recording degradation and neuronal variance observed in chronic intracortical recordings. Many studies reported that the decline of MFR and NIU is to be associated with decreased decoding performance (Wang et al., 2014; Kim and Kim, 2016). We simulated the spike signals to mimic the decline of MFR and NIU, respectively.

Our results showed that the performance of decoders decreased as either MFR or NIU declined. As MFR decreased, the amplitude of neural signals carrying kinematic information got smaller, leading to a drop in signal-noise ratio (SNR). The decline of MFR made it more difficult to get the mappings from firing rate to kinematic data. NIU experiment demonstrated the main consequences of dropping NIU. The first is the loss of neural information. The decrease of NIU reduced the amount of neuron information, resulting in inaccurate prediction (Kim et al., 2018). The second is the change in PDs distributions. The dropping units led to non-uniform PDs distribution, which induced instability in decoding performance. Therefore, the prediction speed had a large range of bias to a certain speed (Chase et al., 2009), which led to a larger standard error in RMSEs.

Unlike the MFR and NIU experiments, the variations of PDs had little influence on signal quality. The neural firing rate was still highly correlated with kinematic signals. Therefore, there was no obvious decline in retrained decoding results. Nevertheless, the PDs variations changed the mappings from firing rate to kinematic data for each session (Jarosiewicz et al., 2015), causing the declining performance of static decoding.

### Comparison between different decoders in chronic decoding

Previous studies have reported RNN as having either better or similar performance compared to OLE and KF (Sussillo et al., 2012; Ahmadi et al., 2021), which is supported by the result of this study. In the first few sessions, where the signal quality was high and the firing patterns were similar, the non-linear recurrent structure was able to ensure better performance of RNN. However, these advantages could not ensure RNN’s performance when the signal quality decreased and the firing patterns changed in the latter sessions, resulting in decreasing performance. Specifically, for RNN under retrained decoding, the neural mappings in the training and testing dataset were the same, thus minimizing the influence of different sessions’ non-stationarity. Moreover, the recurrent layer in RNN could extract speed information from previous input data, which helped with decoding. RNN performed best when the spike signal degradation was slight and performed similarly to the other two decoders when the degradation was severe, indicating that RNN had more outstanding decoding ability than KF and OLE.

In addition, KF outperformed OLE in most scenarios due to its utilization of the Markov model and the linear transform function. The Kalman gain also offered error correction in the prediction process. Whereas OLE only used a linear transformation matrix and had no iteration step (Chase et al., 2009). The performance of OLE relied heavily on the quality of training data, thus causing obvious bias if the neural signals contained substantial noise. Therefore, static OLE might perform better than retrained OLE when there was severe degradation in signal quality, as shown in Figure 5. Overall, RNN performed better than linear decoders such as KF and OLE, and KF outperformed OLE due to the iteration step with Kalman gain.

### Comparison between two training schemes in chronic decoding

In chronic recording, the performance of decoders under static scheme got worse when non-stationarity accumulated. Previous studies have reported the performance advantage of retrained scheme over static scheme (Jarosiewicz et al., 2013; Ahmadi et al., 2021). This study reaffirms this conclusion as our retrained RNN and KF almost always outperformed static ones when only a single type of neuronal non-stationarity was introduced.

In the first few sessions of MFR and NIU experiments, the neuron MFR was high and the NIU was sufficient. Hence, no obvious difference was observed between the two schemes due to the high similarity in neural signal patterns and the high SNR. However, in the last few sessions, neural patterns were sufficiently different from the patterns of the static model. For both schemes, neural signal degradation made it difficult to capture the neural patterns, which led to performance drops. In PD experiment, both schemes achieved similar performance in the first few sessions when the neural pattern remained relatively stable. In the last few sessions, the accumulation of PDs variances changed the mapping from neural signal to kinematic data, causing the performance decline in static scheme. On the other hand, retrained scheme showed stable performance since the SNR was high despite changes in PDs.

### Comparison to previous studies

Previous studies have investigated the effects of neuronal ensemble properties on decoders with spike simulation (Kim and Kim, 2016; Kim et al., 2018). In our study, we further investigated the neuronal properties in the case of chronic recordings and simulated the non-stationarity variations based on previous studies (Wang et al., 2014; Wan et al., 2019). Furthermore, we introduced RNN and two training schemes in our performance comparison, making the investigation more comprehensive. We also used the PV simulation model (Inoue et al., 2018) and real kinematic signal from monkey experiments.

It was reported that RNN had better performance and was more robust than KF in close-loop experiments (Sussillo et al., 2012; Ahmadi et al., 2021). Using actual neural signals with high quality, RNN showed robust and prominent performance, which aligns with our result of the first few testing sessions. However, when RNN was put under greater neural signal degradation, we found that when the trajectory information was covered by noise signal, all decoders will experience a drop in performance. This degradation of signal quality has a particularly great impact on the performance of decoders under static scheme, thus in last few sessions, static RNN performed similar to KF.

As for spike simulation, various spike simulations based on different methods have been applied in previous studies, such as PV model (Chase et al., 2009; Inoue et al., 2018; Wan et al., 2019), deep neural network (Ramesh et al., 2019) and large datasets based simulation (Hosman et al., 2019). As a powerful tool in neuroscience research, simulation can provide neural signals with specific non-stationarity for decoder validation or chronic recording investigation. It removed the influences of uncertain noise in actual neural signals and enabled us to focus on the research problems. It is challenging to implement neuronal properties variation to the simulation models based on generative neural networks and existing large datasets since these two simulation methods do not come from the observation of physiological experiments and are thus less explainable from a neuroscience perspective.

### Limitations and future work

Although the effects of neural signal non-stationarity on decoding performance were investigated with the simulation datasets, the real-world situations are more complicated than what was demonstrated in our simulation work. The parameters adopted for the ranges of MFR, NIU, and PD variation were relatively arbitrary, which is the main limitation of our simulation work. Therefore, we propose two possible directions for future improvements. Firstly, due to the simple and arbitrary nature of chronic simulation models, the change tendency can only be shown qualitatively over time. In the future, a more detailed and complex model could be used to simulate the neural signal degradations and variances. For instance, a changing model could be extracted from actual recording data with curve-fitting methods. Secondly, more non-stationarity metrics can be included to describe the chronic variation of spike signals. The non-stationarity metrics in chronic intracortical recordings are not limited to those investigated in this study. The neural non-stationarity can be explained more exhaustively if more metrics are considered.

## Data availability statement

The original contributions presented in this study are included in the article/Supplementary material, further inquiries can be directed to the corresponding author.

## Ethics statement

This animal study was reviewed and approved by the Animal Care Committee at Zhejiang University.

## Author contributions

ZW finished the code for simulation, decoding, result analysis, and writing. XR and TL provided the code for RNN. PL improved the writing quality and revised the manuscript critically. WC and SZ contributed to the manuscript writing, experiment supervision, and funding acquisition. All authors contributed to the article and approved the submitted version.

## Supplementary material

Supplementary Figure 1 The figure of neural firing rate statistics. We ranked the firing rate of neurons from the motor cortex of a monkey performing 2D center-out task. Each bar represents a firing rate and there are 88 neurons. The red line shows the 28 Hz firing rate.
