# Supplementary material for: The influence of non-stationarity of spike signals on decoding performance in intracortical brain-computer interface: a simulation study
Source: Front Comput Neurosci. 2023 May 12;17:1135783. doi: 10.3389/fncom.2023.1135783 (PMC10213332; doi:10.3389/fncom.2023.1135783)
Supplement: Supplementary file 1 [file Table_1.DOCX]

Supplementary Table.1 The Group-Level two-way ANCOVA Analysis

| Experiments | Group | static | | retrained | |
| --- | --- | --- | --- | --- | --- |
|  |  | CC | RMSE | CC | RMSE |
| MFR | decoder | 0 | 0 | 0 | 0 |
|  | metric | 0 | 0 | 0 | 0 |
|  | interaction | 0.013 | 0 | 0.509 | 0.002 |
| NIU | decoder | 0 | 0 | 0 | 0 |
|  | metric | 0 | 0 | 0 | 0 |
|  | interaction | 0.071 | 0.080 | 0.016 | 0 |
| PD | decoder | 0 | 0 | 0 | 0 |
|  | metric | 0 | 0 | 0 | 0 |
|  | interaction | 0.175 | 0.133 | 0 | 0 |

Supplementary Table.1. The p-value of two-way ANCOVA. The dependent variable are the decoding results (CC and RMSE). The independent variables are the decoder types and the non-stationarity metrics. The p-value are recorded as 0 if p < 0.001.
