# Supplementary figures and images for: The influence of non-stationarity of spike signals on decoding performance in intracortical brain-computer interface: a simulation study
Source: Front Comput Neurosci. 2023 May 12;17:1135783. doi: 10.3389/fncom.2023.1135783 (PMC10213332; doi:10.3389/fncom.2023.1135783)

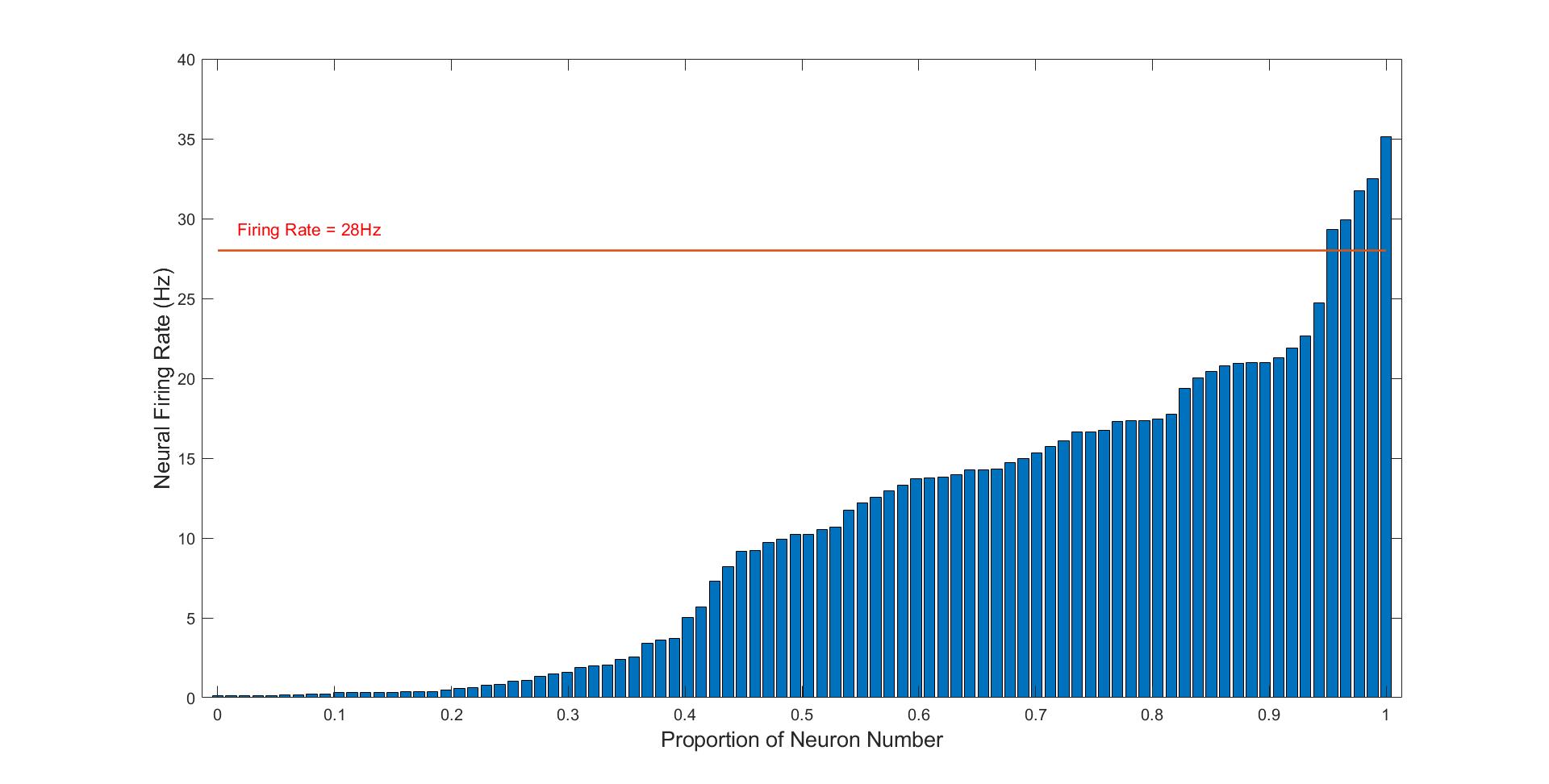

Supplement: Supplementary Figure 1 — The figure of neural firing rate statistics. We ranked the firing rate of neurons from the motor cortex of a monkey performing 2D center-out task. Each bar represents a firing rate and there are 88 neurons. The red line shows the 28 Hz firing rate. [file Image_1.JPEG]
